# Superficial vein thrombosis of the upper extremity after pectoralis major repair: An uncommon complication

**DOI:** 10.1016/j.tcr.2026.101351

**Published:** 2026-04-26

**Authors:** Bahaaldin Helal, Patrick J. Carroll, Sultan Alsuhaim, Matthew Nakouzi, Amr Elmaraghy

**Affiliations:** a Temerty Faculty of Medicine, University of Toronto, Toronto, Ontario, Canada; b Division of Orthopaedic Surgery, Department of Surgery, University of Toronto, Toronto, Ontario, Canada; c St. Joseph's Health Centre, Unity Health Toronto, Toronto, Ontario, Canada; d Li Ka Shing Knowledge Institute, 209 Victoria St., Toronto, Ontario, Canada; e Department of Orthopedic Surgery, Prince Sultan Military Medical City, Riyadh, Saudi Arabia; f Royal College of Surgeons in Ireland, Dublin, Ireland

**Keywords:** Pectoralis major repair, Superficial vein thrombosis, Upper extremity, Cephalic vein thrombosis, Postoperative complication

## Abstract

**Background:**

Superficial venous thrombosis (SVT) is traditionally considered a benign condition, yet recent evidence indicates there may be a clinically significant overlap with it and deep venous thrombosis (DVT) and/or pulmonary embolism (PE) in a significant proportion of cases. Despite the well-established low incidence of venous thrombo-embolism (VTE) following upper extremity orthopaedic procedures such as pectoralis major (PM) tendon repair, thrombotic complications remain clinically noteworthy and potentially serious.

**Case presentation:**

A 43-year-old active male presented with left shoulder pain following a skiing accident, and he was diagnosed clinically with a complete PM tendon rupture. Surgical repair was performed uneventfully eight weeks after injury without pharmacologic thromboprophylaxis, consistent with guidelines for low-risk upper extremity surgeries. Ten days postoperatively, the patient developed a painful, cord-like swelling along the ipsilateral cephalic vein. Duplex ultrasound confirmed an isolated thrombosis within the cephalic vein extending proximally along the arm. The patient was initiated on therapeutic anticoagulation with rivaroxaban, resulting in rapid symptomatic improvement and an uncomplicated recovery.

**Conclusion:**

This case underscores the importance of maintaining clinical vigilance for superficial vein thrombosis even in surgical contexts considered low risk for VTE. Prompt imaging to differentiate SVT from DVT is crucial, and anticoagulation therapy may be warranted in cases involving extensive superficial thrombosis or proximity to deep venous systems. Appropriate patient education, timely recognition, and tailored treatment strategies are key steps toward ensuring optimal outcomes.

## Introduction

Superficial venous thrombosis (SVT) refers to thrombosis in a superficial vein. This condition is traditionally viewed as a benign localized process. However, emerging evidence shows SVT can overlap with deep vein thrombosis (DVT) and pulmonary embolism (PE) more often than previously thought. In a meta-analysis of lower-extremity SVT, Di Minno et al. [1] found concomitant DVT in 18.1% of patients and PE in 6.9%, indicating a significant minority have a simultaneous deep venous clot. The authors concluded that SVT should not be dismissed without evaluation—selective screening for DVT/PE is warranted, as SVT may coexist with or herald a dangerous deep clot.

Pectoralis major (PM) repair is a surgery performed to reattach the torn tendon to its footprint in the proximal humerus, occurring most often in young athletic males after injuries sustained during weightlifting, especially bench press [2]. Unlike major hip or knee operations, upper extremity surgeries like PM tendon repairs have a very low incidence of postoperative venous thromboembolism (VTE). Lower extremity orthopaedic procedures are well known for high VTE risk (historically, 45–50% incidence of DVT without prophylaxis in some contexts), prompting routine chemical thromboprophylaxis in those patients [3], [4]. In contrast, the relationship does not hold true for upper extremity surgeries. VTE events after shoulder and arm procedures are exceedingly uncommon with one study looking at 3357 upper extremity orthopaedic cases reporting only 6 VTE events total (approximately 0.18% incidence) [5]. Similarly, a recently published meta-analysis on PM repairs showed that the incidence rate of VTE events post-operatively is 0.36%, reflecting how uncommon DVT/PE are in this setting [2]. Due to this low risk, routine pharmacologic thromboprophylaxis is typically not recommended after upper extremity procedures, as the potential bleeding risks generally outweigh the benefits of thrombosis prevention in this patient population. Instead, standard postoperative care focuses on early mobilization and mechanical measures for general VTE prevention. Clinical guidelines support this approach—the International Consensus Meeting on Venous Thromboembolism (ICM-VTE) guideline notes that VTE prophylaxis is generally not needed for upper extremity surgery (especially if done under regional anesthesia or short duration), and should only be considered in prolonged cases or patients with added risk factors [6].

Given the rarity of VTE in upper extremity procedures, a thrombotic complication following PM tendon repair has not been reported previously in the literature, making this case noteworthy. In this report, we present a case of cephalic venous thrombosis following PM tendon repair in a healthy, relatively young, athletic male.

## Case presentation

A 43-year-old right-hand-dominant man who works in sales (and is an active recreational soccer and tennis player) presented with left shoulder pain and noticeable chest asymmetry for three weeks following a skiing accident. He denied any prior corticosteroid injections or direct shoulder injuries. His past medical history was unremarkable. He is a non-smoker, though he reported occasional recreational use of intranasal cocaine and psilocybin (a naturally occurring hallucinogenic substance), with no use in the two weeks leading up to surgery.

Clinical examination revealed abnormal anterior axillary fold on the left and visible pectoral asymmetry compared to the uninjured side. He had a positive PM Index test (suggesting a structurally significant disruptive injury) and demonstrated weakness in shoulder adduction when the arm was positioned in abduction and external rotation [7]. These findings were consistent with a full-thickness rupture of the left PM tendon. In our practice, this diagnosis is made clinically; imaging such as MRI is not routinely required in an acute presentation with clear exam findings.

Routine preoperative blood work and coagulation profile were within normal limits. The patient underwent surgical repair approximately eight weeks post-injury. The procedure was done under general anesthesia combined with an interscalene regional block for postoperative pain control. The patient was positioned in a beach-chair position. Prophylactic antibiotics 2 g of cefazolin and 1 g of tranexamic acid were administered prior to the incision. Following surface anatomy marking and skin infiltration with 1:100,000 epinephrine mixed with local anesthetic, a deltopectoral approach was used. The cephalic vein was identified and carefully preserved, retracting it laterally with the deltoid throughout the exposure. The torn tendon was identified as a chronic tear (timing), at or within the musculotendinous junction (location), full thickness and complete width (extent), thus leading to its classification as a C/2/FC tear [8].

After achieving adequate muscle mobilization, six #2 braided high strength non-absorbable sutures (FiberWire and TigerWire, Arthrex, Naples, Fl.) were placed, two for each upper, middle, and lower anchor points. Using retractors, the biceps tendon was retracted medially, footprint exposed and scarified for healing. Three unicortical 3.2 mm drill holes were spaced to correspond with the suture pairs, and PEC buttons (Arthrex, Naples, Fl.) were loaded and deployed. With the arm in adduction and neutral rotation, the sutures were tied sequentially from caudal to cranial, securing the tendon to its anatomical footprint. The repair restored the anterior axillary contour without undue tension. The Brachio-Pectoral fascia and the fascia between the deltoid and PM were repaired using 2–0 braided absorbable suture (Polysorb), keeping the cephalic vein deep and intact. Skin closure was performed in layers using interrupted 3–0 braided absorbable suture (Polysorb) and running subcuticular 3–0 monofilament absorbable suture (Biosyn), reinforced with adhesive bandages (Steri-Strips). The arm was placed in a sling for comfort and protection of the repair.

**Fig. 1:**
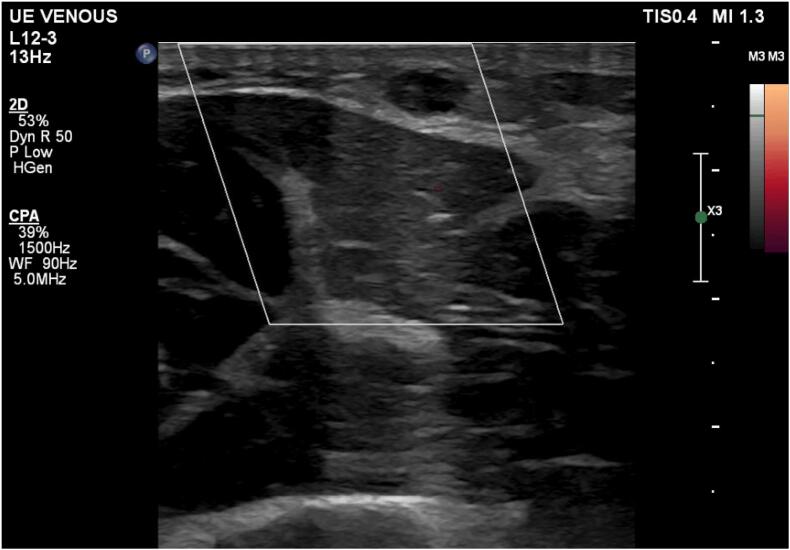
Duplex ultrasound image of the left arm showing a superficial vein thrombosis in the cephalic vein.

At his 10-day postoperative follow-up, the patient presented with a painful, cord-like swelling in the ipsilateral proximal forearm, along the course of the cephalic vein. There was no swelling or edema in his arm. An urgent duplex ultrasound revealed an occlusive vein thrombosis in the cephalic vein, extending from the forearm into the upper arm (Fig. 1). Hematology was consulted, and the patient was started on rivaroxaban 15 mg orally twice daily for 21 days, followed by 20 mg once daily for a total treatment duration of 10 weeks.

Within a few days of starting anticoagulation, the patient reported symptom improvement and proceeded with his rehabilitation protocol without any complications.

## Discussion

PM tendon rupture is a rare injury and VTE events following upper extremity surgery are exceedingly uncommon, which made this case particularly unusual [5]. In this patient, a thrombus developed in the cephalic vein following an otherwise uneventful PM tendon repair. The cephalic vein thrombosis presented clinically as a palpable cord with pain in the forearm, which is a classic presentation for superficial thrombophlebitis [9]. Ultrasound confirmed that the clot in the cephalic vein extended upward more than 5 cm. Although the cephalic vein itself is superficial, it drains into the axillary vein near the shoulder. Thrombosis in a superficial vein close to where it enters the deep venous system can effectively behave like a DVT [9], [10]. In this case, the decision was made to treat with full-dose anticoagulation due to the extent and location of the thrombus, to prevent propagation into the axillary-subclavian deep system and/or a potential PE. This management is consistent with current recommendations for extensive SVT or those at junctions: if the clot is extensive or close to a deep vein, anticoagulation is indicated to minimize progression to DVT/PE [9], [10], [11]. Our patient's prompt improvement on rivaroxaban and uneventful recovery support the effectiveness of this approach.

The cause of this patient's cephalic vein thrombosis is likely multifactorial. Unlike the lower extremity, the upper extremity is not routinely considered high-risk for stasis or thrombosis after surgery. He did not have an indwelling central line or obvious compression that could precipitate an upper extremity DVT [12]. However, the surgical procedure itself could have contributed local factors: dissection and retraction around the cephalic vein might have caused endothelial injury or spasm. A study looking at cephalic vein patency and limb swelling during and after shoulder arthroplasty showed medial cephalic vein mobilization in a deltopectoral approach resulted in significantly less arm edema compared to lateral vein mobilization/ligation [13]. In PM repair, the cephalic vein is routinely brought laterally to isolate the PM tendon and specifically the upper border of the clavicular head, in order to determine if it is intact or torn. However, retractor usage during the insertion of the torn tendon could cause localized trauma to the cephalic vein as shown in this study which may contribute to increased VTE risk in PM repair. This would need to be further studied before any definitive conclusion could be made in PM repair and cephalic vein direction. Postoperatively, the patient's arm was immobilized in a sling, which could lead to venous stasis in the superficial veins of the dependent arm—especially in the context of limited muscle pumping activity while in a sling. It is also notable that tranexamic acid (TXA) was used intraoperatively to reduce bleeding; while TXA has not been definitively shown to increase thrombosis risk in orthopaedic surgery, theoretically it promotes clot stability and, in a patient who ultimately developed a clot, one might question if it played a minor role [14], [15]. Additionally, cocaine, a recreational drug occasionally used by the patient, has been associated with increased thrombogenic risk due to its vasoconstrictive properties and potential to induce endothelial dysfunction, further complicating the patient's postoperative thrombosis risk profile [16], [17], [18]. Nevertheless, no strong predisposing risk factors were clearly identified—this thrombotic event appears to be an idiosyncratic, surgery-associated complication in an otherwise low-risk individual.

A review of the literature finds very few reported cases of upper-extremity SVT associated with PM injuries or surgery. Pozder et al. [19] described a case involving a young male who sustained a traumatic PM rupture following a motor vehicle accident and was concurrently diagnosed with cephalic vein thrombosis; the patient received anticoagulation therapy with heparin/low molecular weight heparin (LMWH) prior to PM surgical repair. In contrast, our case involved thrombosis occurring postoperatively rather than at the time of initial injury. Upper extremity thrombosis in general is uncommon and usually associated with indwelling catheters or repetitive stress (effort thrombosis in athletes) [12]. In the orthopaedic postoperative context, upper extremity VTE incidence is 0.18% [5]. To our knowledge, this is the first reported case of SVT following PM tendon repair. Given the senior author's extensive experience with several hundred PM repairs, this represents his first known case of VTE following this procedure.

This case highlights a few clinically important points. Firstly, even in surgeries with very low expected VTE risk, clinicians should remain vigilant about unusual thrombotic presentations. A complaint of a tender palpable cord in the upper extremity, even without diffuse swelling, should raise the suspicion of VTE and prompt evaluation with ultrasound, as superficial thrombosis can be the first sign of a potentially significant thrombosis. Secondly, superficial vein thrombosis should not be automatically dismissed as benign—especially if the thrombus is extensive or near deep vein connections. In such cases, as demonstrated here, treating the patient with anticoagulation is prudent to prevent progression or embolization. Finally, although current guidelines generally do not recommend routine chemical prophylaxis in low-risk upper extremity surgeries, selective preventative measures—such as postoperative aspirin and mechanical prophylaxis—have been adopted by certain institutions [20]. This variability highlights the potential clinical significance of thrombotic events even following procedures traditionally considered low-risk. As such, postoperative protocols should emphasize patient education on early signs of thrombosis and timely follow-up. A concise summary of clinical signs of SVT is provided in Table 1 to aid clinicians and surgeons in early recognition and timely intervention. In patients with any unusual symptoms, early duplex scanning can facilitate prompt diagnosis, appropriate consultation, and definitive treatment.

**Table 1:** Clinical signs of superficial venous thrombosis.

Clinical signs	Presentation	Evidence
Palpable tender cord	A distinct, palpable, firm, and tender “cord” along the path of a superficial vein (e.g., cephalic, basilic). This is a classic and highly suggestive finding.	[9], [21], [22], [23], [24]
Erythema ± warmth	Linear erythema (redness) and warmth directly overlying the path of the inflamed vein. Skin discoloration may also be present.	[9], [22], [23], [24], [25]
Edema	Localized, often minimal, or absent. May present as a firm, linear unilateral swelling directly along the vein's course.	[22], [23], [24], [25]

## Conclusion

We present an unusual case of a superficial vein thrombosis occurring after PM tendon repair in a healthy, active 43-year-old male. Overall, routine thromboprophylaxis remains unwarranted in PM repairs given the rarity of VTE, but patient-specific risk assessment and education are vital. Despite the baseline risk of VTE in upper extremity orthopaedic surgery being exceedingly low, this case underscores the importance of recognizing and managing SVT when it does occur. Superficial vein clots, particularly when near deep veins, pose a risk of progression into the deep venous system, potentially leading to serious complications. Even in surgeries typically seen as low-risk, clinicians should remain alert and promptly investigate if a patient develops symptoms suggestive of thrombosis. Timely evaluation with ultrasound and appropriate anticoagulation therapy can lead to excellent outcomes, as demonstrated by this patient's full recovery without sequelae.

## CRediT authorship contribution statement

**Bahaaldin Helal:** Conceptualization, Data curation, Writing – original draft. **Patrick J. Carroll:** Supervision, Writing – review & editing. **Sultan Alsuhaim:** Conceptualization, Data curation, Writing – review & editing. **Matthew Nakouzi:** Writing – review & editing. **Amr Elmaraghy:** Conceptualization, Supervision, Writing – review & editing.

## Declaration of competing interest

The authors declare that they have no known competing interests that could have influenced this work.
